# Non-Muscle Myosin IIa Heavy Chain Links Squamous-Cell Carcinoma of the Head and Neck to the DNA Damage Response

**DOI:** 10.3389/fonc.2014.00228

**Published:** 2014-08-25

**Authors:** David R. Raleigh, Daphne A. Haas-Kogan

**Affiliations:** ^1^Department of Radiation Oncology, University of California San Francisco, San Francisco, CA, USA; ^2^Helen Diller Family Comprehensive Cancer Center, San Francisco, CA, USA

**Keywords:** myosin IIa

The incidence of squamous cell carcinoma of the head and neck (HNSCC) reaches as high as 16.5 cases per 100,000 individuals in the United States (1). Early-stage lesions have a favorable prognosis when treated surgically, but approximately two-thirds of HNSCC are diagnosed at an advanced locoregional stage (2). Chemoradiation is the backbone of treatment for the majority of advanced tumors, and is associated with up to 80% long-term survival for patients with positive prognostic features (3). Notably, the success of genotoxic therapy for advanced HNSCC far exceeds non-surgical control rates for the majority of epithelial tumors. Moreover, the primary benefit from chemotherapy for HNSCC is conferred not through a decrease in distant metastases but through enhanced local control (4, 5). As such, significant efforts have been dedicated to elucidating the molecular drivers of HNSCC genesis and identifying the pathways that sensitize these malignancies to the effects of chemoradiation.

Infection with high-risk human papilloma virus (HPV) subtypes has emerged as an important risk factor for HNSCC. Unlike lesions associated with excessive tobacco and alcohol use, *TP53* mutations are rarely found in HPV-positive HNSCC, which is generally associated with a favorable clinical course (6). Despite this association, no targeted therapies against HPV oncogenes are available for the treatment of patients with HNSCC. Similarly, genetic and epigenetic aberrations of the *H-RAS, NOTCH*, PI3K/Akt, *BRCA1*, and TGF-β pathways have also been associated with HNSCC, but targeted agents remain in preclinical stages for HNSCC (2). In contrast, epidermal growth factor receptor (EGFR) inhibition combined with radiotherapy is an effective strategy for HNSCC (7). EGFR is upregulated in approximately 90% of HNSCCs, and expression level is an independent determinant for decreased survival and impaired locoregional control (8, 9). However, EGFR inhibitors as monotherapy have produced only modest clinical outcomes, and survival from HNSCC has not improved in recent decades. Consequently, enhanced understanding of the molecular carcinogenesis of HNSCC is required to facilitate development of new treatments.

Using an *in vivo* RNA interference (RNAi) strategy to screen for driver mutations involved in tumorigenesis, Schramek and colleagues recently reported a novel link between cutaneous squamous-cell carcinoma (CSCC), HNSCC, and impaired DNA damage response (DDR) signaling (10). To do so, a lentivirus-mediated RNAi library was selectively delivered to the surface ectoderm *in utero* under ultrasound guidance in a *TGF-*β*-receptor-II* conditional knockout murine model of CSSC and HNSCC. Selection of multiple independent small hairpin RNAs (shRNAs) in three or more tumors yielded eight potential tumor suppressors. Among these lesions, 40% were enriched with shRNAs against *Myh9*, which encodes non-muscle myosin IIa heavy chain. When conditionally suppressed in mice with endogenous TGF-β signaling, loss of myosin IIa was sufficient for development of CSCC, albeit with a significantly longer latency. By activating the DDR pathway, Schramek et al. further demonstrate that both myosin IIa suppression and small molecule inhibition delay and reduce p53 activation in response to double strand breaks. Consistently, loss of myosin IIa reduces the expression of p53-responsive genes, including *p21, Fas, Bax, Mdm2*, and *14-3-3*σ. Finally, biochemical assays and microscopy both indicate that p53 fails to accumulate and/or remain in the nucleus in the absence of endogenous myosin IIa activity.

These findings suggest a new role for myosin IIa as a tumor suppressor that facilitates the DDR through stabilization of p53 in the nucleus. Although the present data pertain to CSSC and HNSCC, it is intriguing to consider a broader role for myosin IIa in oncogenesis and the DDR. Also notable from this work is the non-invasive, ultrasound-guided method of selective shRNA delivery *in utero*. This novel technique circumvents the immunological and physiological confounders of orthotopic transplantation, and stands to have broad applications for future genetics screens. However, hairpin efficiency is a central component of RNAi and may bias data away from biological reality. Consistent with this limitation, *TP53*, which is frequently mutated in HNSCC, was not identified as a carcinogenic driver in the present study, presumably secondary to hairpin inefficiency.

Despite the abundance of murine data presented by Schramek et al., the pathophysiological relevance of *Myh9* loss for human malignancy remains uncertain. In this regard, approximately 80% of myosin IIa null HNSCCs in humans show concomitant loss of the TGF-β pathway, suggesting that myosin IIa loss may not be a direct oncogenic driver (10). Furthermore, a mere 30–40% of mice with intact TGF-β signaling develop tumors following *Myh9* suppression and only do so after a mean latency of 1 year. Indeed, less than one-quarter of human SCCs and fewer than one-third of human HNSCCs show weak or absent immunolabeling for myosin IIa. Therefore, rather than functioning as a primary oncogenic driver in human malignancy, *Myh9* mutation may play an accessory role in the genesis or progression of certain HNSCC subsets. In sum, it is likely that inactivating mutations of myosin IIa have a low penetrance in human HNSCC, as was suggested by the initial sequencing effort that identified a link between *Myh9* and HNSCC (11). Nevertheless, these results provide compelling evidence that aberrant activity of the DDR is associated with HNSCC and further support the hypothesis that these tumors are both biologically and genetically diverse. Further studies are necessary to establish whether *Myh9* mutation is of robust prognostic significance and if DDR pathways can be manipulated to enhance the efficacy of treatment for HNSCC.

## Conflict of Interest Statement

The authors declare that the research was conducted in the absence of any commercial or financial relationships that could be construed as a potential conflict of interest.
